# Data-set collected during turning operation of AISI 1045 alloy steel with green cutting fluids in near dry condition

**DOI:** 10.1016/j.dib.2020.106215

**Published:** 2020-08-21

**Authors:** Milon Selvam Dennison, Abisha Meji M, Malik Mohamed Umar

**Affiliations:** School of Engineering & Applied Sciences, Kampala International University (Western Campus), Uganda

**Keywords:** Steel alloy, Turning, Peanut oil, Palm oil, ASTM, Green cutting fluid, Taguchi

## Abstract

This work is to explicate the data collected during the turning of AISI 1045 alloy steel components in near dry condition with emulsified cutting fluids prepared from cooking oils such as Palm oil and Peanut oil. The base oils are tested for its relative density, viscosity and flash point following ASTM standards. Highly influencing turning factors are identified and the experiments are planned and arranged using Taguchi's L_27_(3^5^) orthogonal array, the experiments are repeated to reduce the errors. The quality aspect of machined components and the machining interface temperature is observed as the outcomes. The prediction models are created for the experiments through regression analysis.

**Specifications Table**

**Table:** 

Subject area	Industrial and Manufacturing Engineering
Specific subject area	Machining and Machinability of materials
Type of data	Table, graph and chart
How data were acquired	The tool-work interface temperature was acquired with a infrared thermometer (BEETECH MT-4) at the proximity of 1 ft distance and the quality aspect of turned samples was tested using a surface roughness tester (Mitutoyo SURFTEST SJ201)
Data format	Raw, analyzed
Parameters for data collection	Highly influencing turning factors are identified such as Spindle speed (rpm), Feed (mm/rev), Depth of Cut (mm), Tool corner radius (mm) and Cutting fluids
Description of data collection	Machining of the AISI 1045 steel components was performed following the above said control factors in a high-speed CNC Lathe, the interface temperature was tested while machining, using an infrared thermometer and the quality aspect of the turned steel components were performed using a surface roughness tester.
Data source location	Department of Mechanical Engineering, Kampala International University, Western Campus, P.O. Box 71, Bushenyi-Ishaka, Uganda
Data accessibility	Data are presented within this article and supplementary document.

**Value of the Data**

• The data presented in this article conveys the feasibility of using eco-friendly green cutting fluids in the machining of steel alloy components.
• The data presented in this article such that the preparation of eco-friendly cutting fluids can give a lead to the future researchers in this field.
• The data presented here can be used by researchers in the African continent and even the whole world to compare the machining characteristics of AISI 1045 alloy steel with other edible or non-edible vegetable oil based cutting fluids.
• The data can be used to study the machining characteristics of AISI 1045 alloy steel machined with eco-friendly cutting fluids in near dry conditions.

## 1 Data Description

The vegetable oil based emulsified cutting fluids would ultimately reduce the cost contribution of cutting fluid on the total manufacturing cost and would eliminate the pollution caused by the oil waste fed on the environment [1], [2], [3], [4].

The data explicated in this article is about producing qualitative turned machine components in turning of AISI 1045 alloy steel components with emulsified cutting fluids prepared from eco-friendly natural oils such as Palm oil and Peanut oil. The properties of oils such as relative density, viscosity and flash point are evaluated following ASTM standards. Servocut ‘S’ is the mineral-based cutting fluid used along with the vegetable-based emulsions. The quality aspect of machined components and the machining interface temperature is recorded and presented. The optimum cutting fluid based on quality aspect and tool-work interface temperature controlling aspect is presented in the form of charts.

The Palm oil and Peanut oil were tested for its relative density, kinematic viscosity and flash point, following the test standards ASTM D5355, ASTM D445 and ASTM D92 respectively [1]. The properties of the oil are given in Table 1. An anionic emulsifier is used as an additive for the preparation of water-dispersible oil formulation [1]. The appearance of the emulsifier is a pale yellow to clear viscous liquid with a specific gravity of 0.96. The vegetable-based emulsion formed was homogeneous, stable and did not split during the continuous usage. The cutting fluid compositions are given in Table 2.

**Table 1:** Properties of Oils.

Property	Servocut ‘S’ [5]	Palm Oil	Peanut Oil
	C1	C2	C3
Relative density	0.877	0.924	0.92
Kinematic Viscosity at 40°C	20 cSt	48 cSt	42 cSt
Flash Point	150°C	324°C	315°C

**Table 2:** Cutting fluid composition.

Cutting fluid composition	Servocut ‘S’	Palm Oil	Peanut Oil
	C1	C2	C3
Oil	4%	4%	4%
Additives	1%	1%	1%
Water	95%	95%	95%
**Total**	100%	100%	100%

The highly influencing turning control factors to be spindle speed (n), feed rate (f), depth of cut (d) tool corner radius (r) and cutting fluid (C) are decided for the trials and their levels are indicated in Table 3.

**Table 3:** Turning control factors and levels.

Control factors	Notation	Levels		
		1	2	3
Spindle Speed (rpm)	n	3200	3400	3600
Feed (mm/rev)	f	0.1	0.15	0.2
Depth of Cut (mm)	d	0.1	0.15	0.2
Tool Corner Radius (mm)	r	0.4	0.8	1.2
Cutting fluid (no unit)	C	C1	C2	C3

The quality attribute with the sort of ‘smaller-the-better’ [1, 5] measured in this research work was surface roughness (Ra) of the machined samples and tool-work interface temperature (T) while machining. The Signal-to-Noise Ratio (SNR) for the yield responses was computed by Eq. (1) for each machining condition and the corresponding data are given in Table 4.

(1) $$\text{SNR}=-10\text{lo}g_{10}\left(\frac{1}{n}\sum_{i=1}^{n}{\text{Response}}_{i}^{2}\right)$$

where *i* = 1, 2,…, *n* (here *n* = 5).

**Table 4:** Experimental data.

Exp No.	Control factors					Surface roughness, Ra (µm)					SNR for Ra	Tool-work interface temperature, T ( °C)	SNR for T
	n	f	d	r	c	Trial 1	Trial 2	Trial 3	Trial 4	Mean, Ra			
1	1	1	1	1	1	0.5139	0.6012	0.5598	0.6134	0.5721	4.851	55.6	−34.90
2	1	1	1	1	2	0.4123	0.4521	0.5001	0.4234	0.4470	6.994	56.9	−35.10
3	1	1	1	1	3	0.4003	0.4043	0.4232	0.4103	0.4095	7.754	56.5	−35.04
4	1	2	2	2	1	1.1763	1.0932	1.0733	1.0031	1.0865	−0.720	71.5	−37.08
5	1	2	2	2	2	1.0274	1.1187	1.0686	1.0585	1.0683	−0.574	72.8	−37.24
6	1	2	2	2	3	0.9756	1.1452	1.0452	0.9952	1.0403	−0.343	72.4	−37.19
7	1	3	3	3	1	1.5075	1.6086	1.5675	1.7085	1.5980	−4.072	86.0	−38.69
8	1	3	3	3	2	1.5201	1.5345	1.4991	1.5318	1.5214	−3.645	87.3	−38.82
9	1	3	3	3	3	1.6742	1.7141	1.6932	1.6941	1.6939	−4.578	86.9	−38.78
10	2	1	2	3	1	0.5105	0.5695	0.5654	0.5765	0.5555	5.107	90.4	−39.12
11	2	1	2	3	2	0.5151	0.5253	0.5097	0.5232	0.5183	5.708	91.7	−39.25
12	2	1	2	3	3	0.5801	0.5798	0.5913	0.5801	0.5828	4.689	91.3	−39.21
13	2	2	3	1	1	1.0149	1.0159	1.0258	1.0154	1.0180	−0.155	91.8	−39.25
14	2	2	3	1	2	0.9809	0.9811	0.9789	0.9913	0.9831	0.148	93.1	−39.38
15	2	2	3	1	3	1.0851	1.0952	1.1952	1.0095	1.0963	−0.798	92.7	−39.34
16	2	3	1	2	1	1.4138	1.4247	1.4563	1.3947	1.4224	−3.060	104.9	−40.41
17	2	3	1	2	2	1.3277	1.4178	1.3188	1.3366	1.3502	−2.608	106.2	−40.52
18	2	3	1	2	3	1.5127	1.5029	1.5167	1.4991	1.5079	−3.567	105.8	−40.49
19	3	1	3	2	1	0.4279	0.4179	0.4389	0.4187	0.4259	7.415	113.6	−41.11
20	3	1	3	2	2	0.6172	0.6089	0.6061	0.6243	0.6141	4.235	114.9	−41.21
21	3	1	3	2	3	0.6163	0.6772	0.6972	0.6873	0.6695	3.485	114.5	−41.17
22	3	2	1	3	1	0.8933	0.8912	0.8793	0.8913	0.8888	1.024	132.4	−42.44
23	3	2	1	3	2	0.8714	0.8752	0.8743	0.8712	0.8730	1.179	133.7	−42.52
24	3	2	1	3	3	0.9217	0.9219	0.9301	0.9106	0.9211	0.714	133.3	−42.49
25	3	3	2	1	1	1.2694	1.2534	1.2614	1.2643	1.2621	−2.022	144.0	−43.17
26	3	3	2	1	2	1.3775	1.3624	1.3914	1.3715	1.3757	−2.770	145.3	−43.25
27	3	3	2	1	3	1.2915	1.3004	1.2904	1.2913	1.2934	−2.235	144.9	−43.22

The F-Test and P-test are led dependent on the responses and the control factors [6]. Tables 5 and Table 6 show the outcomes accomplished by ANOVA. The regression value is seen as under 0.05 for both response factors demonstrating that the created model is at 95% of the confidence limit [1, 5]. The P-value is determined by 95% of the confidence limit. The P-value under 0.05 shows the noteworthy impact of the control factors on the responses.

**Table 5:** ANOVA for surface roughness (Ra).

Source	DF	Seq SS	Contribution	Adj SS	Adj MS	F-Value	P-Value
N	1	0.06887	1.70%	0.00006	0.000056	0.02	0.899
F	1	3.76321	92.88%	0.18195	0.181946	53.90	0.000
D	1	0.08380	2.07%	0.00063	0.000631	0.19	0.672
R	1	0.02689	0.66%	0.00110	0.001098	0.33	0.577
C	1	0.00825	0.20%	0.02276	0.022764	6.74	0.021
Nf	1	0.01347	0.33%	0.00384	0.003840	1.14	0.304
Nd	1	0.00001	0.00%	0.00052	0.000522	0.15	0.700
Nc	1	0.01471	0.36%	0.01471	0.014705	4.36	0.056
Fd	1	0.00290	0.07%	0.00290	0.002903	0.86	0.369
Fc	1	0.00090	0.02%	0.00090	0.000904	0.27	0.613
Dc	1	0.01783	0.44%	0.01783	0.017829	5.28	0.037
Rc	1	0.00362	0.09%	0.00362	0.003624	1.07	0.318
Error	14	0.04726	1.17%	0.04726	0.003375		
Total	26	4.05173	100.00%

**Table 6:** ANOVA for tool-work interface temperature (T).

Source	DF	Seq SS	Contribution	Adj SS	Adj MS	F-Value	P-Value
n	1	15,646.8	82.45%	20.7	20.69	3.01	0.105
f	1	2835.0	14.94%	15.3	15.31	2.23	0.158
d	1	1.1	0.01%	27.9	27.85	4.06	0.064
r	1	151.4	0.80%	88.6	88.62	12.91	0.003
c	1	3.6	0.02%	0.1	0.15	0.02	0.886
nf	1	189.6	1.00%	67.6	67.58	9.84	0.007
nd	1	5.1	0.03%	27.8	27.80	4.05	0.064
nc	1	0.0	0.00%	0.0	0.00	0.00	1.000
fd	1	48.7	0.26%	48.7	48.66	7.09	0.019
fc	1	0.0	0.00%	0.0	0.00	0.00	1.000
dc	1	0.0	0.00%	0.0	0.00	0.00	1.000
rc	1	0.0	0.00%	0.0	0.00	0.00	1.000
Error	14	96.1	0.51%	96.1	6.87		
Total	26	18,977.4	100.00%				

### 1.1 Prediction Model

By means of regression analysis with the aid of MINITAB 17 statistical software, the effect of turning factors on the responses was modeled and presented in Eq. (2) and (3).

(2) $$\begin{array}{ccc} \mathrm{Ra} & = & 0.129-0.018n+0.5825f+0.060d-0.0282r-0.1778c-0.0468\text{nf}\\ & & -\;0.0173\text{nd}+0.0350\text{nc}-0.0288\text{fd}+0.0087\text{fc}+0.0385\text{dc}+0.0174\text{rc} \end{array}$$

(3) T=40.95+10.95n−5.34f−12.57d+8.00r+0.45c+6.21nf+3.99nd+3.73fd

For the above mathematical model, it was found that r^2^ = 0.98 for surface roughness and r^2^ = 0.99 for tool-work interface temperature, where ‘r’ is the correlation coefficient and the value range of ‘r^2^’ should be between 0.8 and 1 [7].

**Fig. 1:**
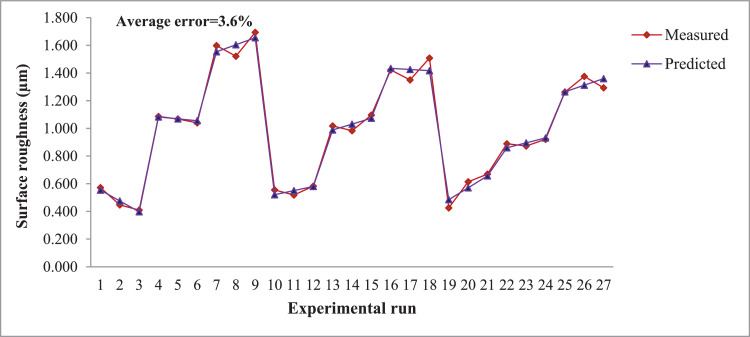
Regression model for surface roughness.

**Fig. 2:**
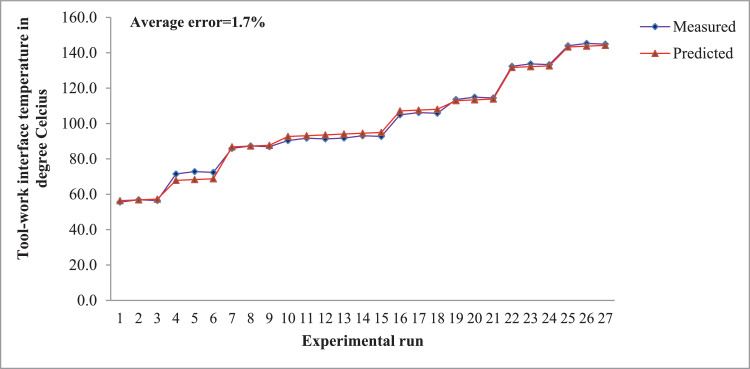
Regression model for tool-work interface temperature.

The predicted runs have very close values with the measured data. For reliable statistical analyses, error values must be smaller than 20% [8]. The regression model data is detailed in the form of graph for both the outcomes such as surface roughness and tool-work interface temperature and are presented in Fig. 1, Fig. 2 respectively.

### 1.2 Effectiveness of cutting fluids

**Fig. 3:**
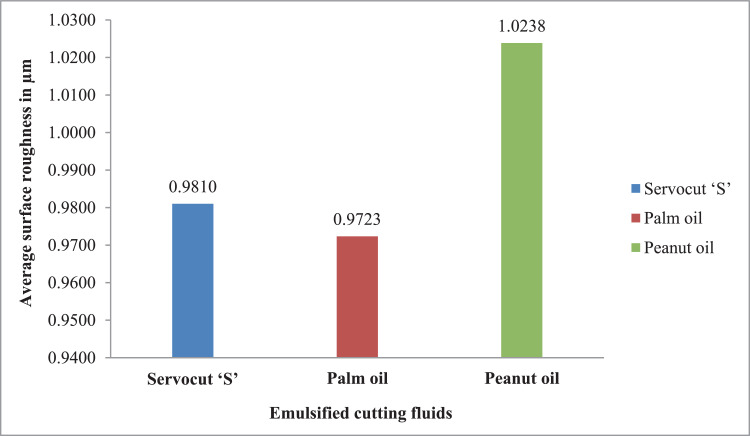
Effectiveness of cutting fluids for average surface roughness.

**Fig. 4:**
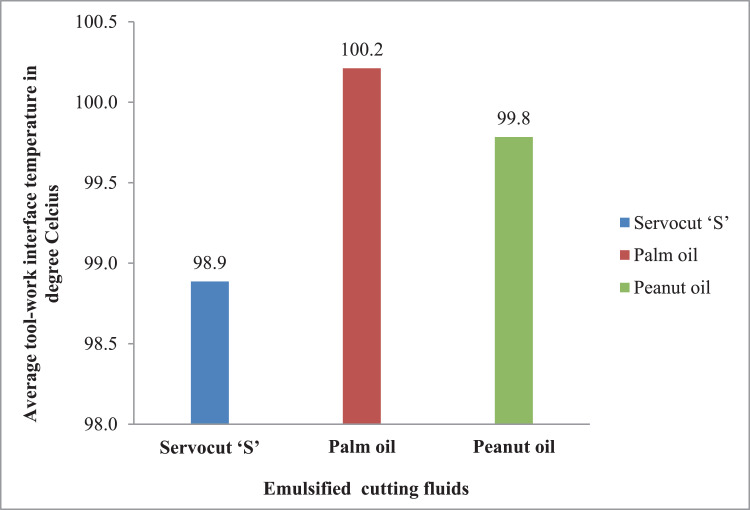
Effectiveness of cutting fluids for tool-work interface temperature.

The effectiveness of cutting fluids used in this research work and the average data of surface roughness and tool-work interface temperature are observed and depicted in Fig. 3, Fig. 4. The raw data associated with the Fig. 3 and Fig. 4 can be found in the supplementary file.

## 2 Design, materials, and methods

The experiments were arranged in view of Taguchi's orthogonal array in a CNC turning center (LMW Smart Junior). The turning operation is done on AISI 1045 cylindrical components of (ϕ50mm x 120 mm) by utilizing PCLNR tool holder and CNMG diamond finishing titanium nitride of three different tool corner radius such as 0.4 mm, 0.8 mm and 1.2 mm. Emulsified cutting fluids prepared of mineral oil, palm oil and peanut oil are used as the coolants/lubricants in this research. All through the experimentation, a steady flow rate of emulsified cutting fluids at the rate of 44.8 ml/hr and steady pressure of 5 bar was kept for the near dry cooling system. While turning the steel samples, the tool-work interface temperature was measured using an infrared thermometer (BEETECH MT-4) at the proximity of 1 ft distance. The quality aspect of turned samples was tested using a surface roughness tester (Mitutoyo SURFTEST SJ201).

## Declaration of Competing Interest

The authors declare that they have no known competing financial interests or personal relationships that could have appeared to influence the work reported in this article.
